# Supplementary material for: Structural equation modelling of the role of cognition in functional interference and treatment nonadherence among haemodialysis patients
Source: PLoS One. 2024 Oct 17;19(10):e0312039. doi: 10.1371/journal.pone.0312039 (PMC11486361; doi:10.1371/journal.pone.0312039)
Supplement: Attachment — Submitted filename: Response to Reviewers.docx [file pone.0312039.s002.docx]

Dear Editor and Reviewers,

We would like to thank you for the letter dated 17^th^ Sep 2024 and the opportunity to resubmit our revised manuscript (PONE-D-24-17737 “Structural equation modelling of the role of cognition in functional interference and treatment nonadherence among haemodialysis patients”) to PLOS ONE. We would like to thank the reviewer for the positive feedback and comments which helped improving this manuscript.

In this revision letter, we have responded to the reviewer comments individually, indicating how we addressed each concern and describing the changes we have made. The comments have been appended alongside this letter, with our responses attached below each comment. In the manuscript, all changes have been highlighted in red font.

With kindest regards,

Konstadina Griva, Corresponding Author (on behalf of all authors)

Associate Professor of Health Psychology and Behavioral Medicine

Lee Kong Chian School of Medicine, Nanyang Technological University

***************************************************************************

**Reviewer 1 Comment 1:** First, the authors applied the SEM model to demonstrate that subjective cognitive complaints and everyday problem-solving skills appear to be stronger predictors of functional outcomes compared to objective performance. However, as a cross-sectional study, it's hard to determine the cause-and-effect relationship between two or more variables, a kind of association in the results couldn’t easily be explained with one variable being the cause and the other one being the outcome. It is also not proper to regard functional interference and treatment nonadherence as a certain kind of outcome, which is also commonly used in a cohort study, so the expressions in the manuscript should be adjusted to avoid misunderstanding.

**Authors’ response and actions:** Thank you for your comment. We have decided to revise our manuscript to use the term “dependent variable” rather than “outcome” in the main text where possible. This change aligns with the nature of our cross-sectional study, which is designed to explore associations rather than infer causality.

Where appropriate we have avoided using terms like “effect” or “predict”, and have changed these terms to “association” or “relation”.

We have also taken your advice to ensure that the limitations of our study design are clearly communicated. In the discussion section, we have added the following points to highlight the nature of our cross-sectional analysis and the need for caution:

- “First, it should be noted that this is a cross-sectional observational study. Also, SEM is not inherently a causal method [21]. Caution is therefore needed when interpreting the significant paths in our final model. Although the directions of these associations have been hypothesised and tested, the study design and statistical method do not permit the interpretation of a cause-and-effect sequence. Future longitudinal studies and intervention research are needed to explore potential causal mechanisms and further validate our observations.”

We hope that these revisions address your concerns and strengthen the clarity and accuracy of our manuscript.

**Reviewer 1 Comment 2:** Also, the results of this study showed that subjective cognitive complaints were related to functional interference and treatment nonadherence, does that mean objective assessment of cognitive impairment is not necessary before the patients have complaints? Generally, we could have a valuable time window between objective and subjective cognitive impairment, which may provide effective interference to the patients.

**Authors’ response and actions:** Thank you for this interesting insight. Given the cross-sectional design of the current study and its focus on the associations with functional and behavioural variables, we do not think the results would be able to answer this question. Although everyday cognitive assessments, especially subjective complaints, are the focus of the paper, we do not think that subjective reports should be used in replacement of objective tools. Instead, we believe that the two tools complement each other and self-report has additive utility beyond objective tools to highlight patients’ everyday difficulties and potential risks for future cognitive decline. Nevertheless, we do agree with the reviewer that a staged identification workflow may be optimal for busy clinical settings where self-reports are used as a first-step screening, followed by more comprehensive tests. In the current study, however, we are reluctant to suggest that objective assessments are not necessary before the presence of complaints, because (1) this suggestion is relatively far-fetched based on the findings, and (2) some patients may have objective impairments but no subjective complaints. We have added the following points to the discussion section:

- “Objective and subjective cognition have therefore been considered as distinct constructs that complement each other with different utility in clinical and research settings. Assessing objective CIs can help establishing diagnosis of CIs and allow for advance care planning, whereas assessing SCCs can help identifying aspects of CIs that have the greatest impact on patients, hence allowing for a more patient-centred approach to managing this debilitating complication [62].”
- “We propose that SCCs should be treated as an outcome equally important as objective CIs, and should be assessed in combination with neuropsychological tests where possible.”

**Reviewer 1 Comment 3:** The MoCA used in this study is only a screening tool for mild cognitive impairments, the author should explain why they didn’t apply the standard diagnosis tests for cognitive impairments.

**Authors’ response and actions:** Dear reviewer, we agree that a comprehensive neuropsychological assessment would be optimal for the current study. However, the tools administered in the study are already quite lengthy and therefore we used the MoCA to reduce patients’ response burden. We have acknowledged this as a limitation in the discussion section:

- “Second, the MoCA used in this study is only a brief screening tool for global cognition. No comprehensive neuropsychological battery or standard diagnostic test was conducted to determine objective cognitive function. This is because the tools administered in this study were already very lengthy and required about 30-40 minutes for each patient to complete. A more comprehensive neuropsychological assessment was therefore not carried out considering patients’ response burden and potential fatigue.”
